# Adjustment challenges and coping strategies of Arab female international university students

**DOI:** 10.3389/fpsyg.2023.1125368

**Published:** 2023-05-18

**Authors:** Thseen Nazir, Ayşe Özçiçek

**Affiliations:** Department of Guidance and Counseling, Faculty of Education, Ibn Haldun University, Istanbul, Türkiye

**Keywords:** adjustment issues, gender issues, coping strategies, international students, academic adjustment, sociocultural adjustment, personal adjustment

## Abstract

**Background:**

Over the past decade, Türkiye has experienced an increasing influx of international students, particularly from various Arab countries. The significant number of Arab international university students has prompted researchers to pay more attention to the experiences of this population in the country. This study examined the adjustment problems experienced by Arab female international university students in Türkiye.

**Method:**

The research has a mixed methods design and includes both quantitative and qualitative studies. In the quantitative study, the International Students Adjustment Scale (ISAS) was used with 373 participants to examine the challenges in which dimensions of adjustment (academic, sociocultural, or personal) were more challenging for the study group. In the qualitative study, semi-structured interviews were conducted with 19 Arab female international university students to gain a deeper understanding of their experiences in the domain that is found the issues are more profound.

**Results:**

Data from the quantitative study revealed that these students experienced similar challenges in each dimension of adjustment; however, problems in the sociocultural domain were much more profound. In line with the quantitative study, the interview questions of the qualitative study were prepared to explore the sociocultural domain and included the pre-arrival expectations, the post-arrival adjustment challenges, and the coping strategies of the study group. The Results of the qualitative study showed that although their prearrival expectations were almost met, they encountered various problems, ranging from cultural differences in daily activities to discrimination. It was also found that many of them were reluctant to ask for help, and to cope with adjustment problems, they usually used maladaptive or dysfunctional coping strategies such as avoidance and isolation.

**Discussion:**

The findings of the quantitative study are in line with the previous studies that the overall adjustment of international students is influenced by different domains that are broadly academic, sociocultural, and personal. Among them, the sociocultural domain is found as the one in the study population that encounters more challenges. The results of the qualitative study support the findings of previous studies related to prearrival expectancies, provide more understanding of adjustment challenges and bring more information about adopted coping strategies.

## 1. Introduction

The importance of developing internationalization strategies has become a rapidly growing phenomenon in response to globalization in political, financial, educational, and other fields and the interdependence of different cultures, societies, economies, and populations. The globalization of the education field requires higher education institutions worldwide to adopt new policies, procedures, and guidelines to attract more international students (Ross et al., 2013; Özoglu et al., 2015; Bradford et al., 2017; Gajwani and Chakraborty, 2021). Over the past two decades, Türkiye has also changed its policies, regulations, and legal arrangements to attract international students to the country (Özoglu et al., 2015). According to the report of the Council of Higher Education (CoHE) on the number of students by nationality in 2021, the number of international students in Türkiye reached approximately 224,000 (Education, 2021).

Data from the Turkish Council of Higher Education showed that the number of students from Arab countries has increased significantly over the past 5 years (Education, 2019). Specifically, the number of Arab students from Syria has increased by 1,060%, Egypt by 865%, Libya by 638%, Yemen by 480%, Jordan by 424%, Somalia by 262%, Iraq by 227%, Palestine by 170%, and Morocco by 145% (Aydin, 2021b). In the 2021–2022 academic year, a total of 98,505 Arab students were enrolled in Turkish universities, including 33,802 women (Education, 2021).

The sociocultural and religious norms strongly influence the deportment of Arab women and cause them to face additional challenges in these areas. For example, studies showed that, in some Arab countries, women are not exposed to coeducation from childhood (Altamimi, 2014) or a mixed-gender environment from childhood, which causes them to face enormous challenges in foreign countries, such as Türkiye, where coeducation is practiced. Often, students are taught by the opposite gender (Altamimi and Nyland, 2010).

Arab female international university students represent a remarkable number of population in Türkiye; therefore, their struggles in the adjustment process can be considered more evident. Moreover, understanding their challenges can guide us to take helpful steps to improve the conditions for newly arrived students. However, a lack of scholarly literature comprehensively examines the adjustment problems of Arab female international university students, especially those who have enrolled in Turkish universities. This study aimed to identify the most challenging adjustment domain for Arab female international university students in Türkiye and to uncover their prearrival expectations, postarrival adjustment challenges, and coping strategies to deal with stress.

The research questions of the study are as follows:

1. What is the most challenging adjustment domain for Arab female international university students in Türkiye?
2. What are the prearrival expectations and postarrival adjustment challenges of Arab female international university students?
3. What are the coping strategies Arab female international university students adopted to deal with distressing adjustment problems?

## 2. Literature review

### 2.1. International students' adjustment

The American Psychological Association (APA, 2020) dictionary defines an adjustment as “a change in attitude, behavior, or both by an individual based on a perceived need or desire for change, especially in response to the current environment or to changing, atypical, or unexpected conditions.” Individuals can be considered well-adjusted when they functionally and healthily meet their needs while responding appropriately to environmental and social demands.

International students are those who move to another country to pursue their education (Shapiro et al., 2014; UNESCO, 2022). Moving from one's home country to a host country can significantly change the environment, and adjusting to unfamiliar sociocultural norms while studying in a new academic environment can lead to a mixture of discomfort and personal development (Ammigan et al., 2022).

International students need to adapt to the host country environment in order to approach harmony, balance, and equilibrium. During the acculturation process, individuals may face adjustment challenges (Baklashova and Kazakov, 2016; Alharbi and Smith, 2018). In recent years, various studies have addressed the difficulties international students face in assimilating, acclimating, and acculturating to new social, cultural, and educational environments (Aldwin and Yancura, 2004; Yan, 2020; Koo et al., 2021). In the case of international students, they are likely to face challenges in different areas of adjustment, namely, academic, personal, and sociocultural domains (Aderi et al., 2013; Firang, 2020).

Academic adjustment can be achieved when students respond successfully to educational demands such as motivation, application, and performance (Baker and Siryk, 1999). It involves meeting course requirements and achieving learning outcomes. International students pursuing their academic careers need to acclimate themselves to the academic atmosphere of the university in order to achieve success and persistence in their studies (Hussain and Shen, 2019).

“Socio-cultural adjustment” refers to an individual's ability to adapt to a new environment (Kwon, 2013). It involves international students developing new competencies and skills to cope with the challenges of daily life in a new cultural environment (Ward and Kennedy, 2001). The process of sociocultural adjustment is influenced by cultural learning and the acquisition of social skills, among other factors (Wilson et al., 2013). This dynamic process can create a harmonious fit between an individual and his or her environment. When an individual's social and cultural environment has changed, it is necessary for the individual to learn and cope with the norms, values, standards, and acceptances of the new society in order to successfully adapt to the sociocultural aspects of the new environment (Baron and Hartwig, 2020).

Personal adjustment occurs when an individual establishes a harmonious relationship with his or her environment. In the context of international students, it is related to the intrapsychic state of students as they adapt to their new environment in the host country; the degree to which they experience psychological distress and whether there are concomitant somatic problems are related to the process of personal adjustment (Baker and Siryk, 1999). Personality type, characteristics, early experiences, personal beliefs, values, attitudes, etc. may determine the pace and direction of their adjustment.

### 2.2. The push-and-pull model

Students choose to study abroad for various reasons, which may be related to adverse conditions in their home country or to the attractiveness and higher standards of the destination country. Scholars often explain student choices using the “push and pull” model, and recently the McMahon model (1992) has been widely used by researchers studying the movement of students across international borders for educational purposes (Hailat et al., 2022). According to McMahon (1992), the “push” or outbound model describes the disadvantageous factors of a home country that cause students to leave and study abroad, while the “pull” or inbound model describes the attractive qualities of the host country. The literature shows that for the Middle Eastern students, the pull factors to Türkiye are its geographical proximity to the Middle East and Europe (Türkiye being a passage to the latter), the supportive policies of the Turkish government toward Middle Eastern students and refugees (Aydin, 2021a), the significant Muslim population in Türkiye (Çetin et al., 2017), and religious compatibility that positively influence student's decision to study abroad (Chang and Chou, 2021). In Addition, factors such as quality of education, affordability, recommendations from others, and scholarship opportunities (Ilhan et al., 2012; Özoglu et al., 2015; Snoubar, 2017; Aydin, 2021b) are also found to be the pull factors for studying in Türkiye.

### 2.3. Acculturation stress

The chosen host country may be attractive, full of opportunities, or culturally and geographically close to the home country; however, students may still experience stress due to encountering a different culture. Several studies indicate that adjusting to a new environment can be a significant source of stress that affects a person's physical and psychological wellbeing (Bae, 2020). Students who encounter a new environment and cultural differences between their home culture and the culture of the country they are studying may experience acculturative stress (Schwartz and Zamboanga, 2008). Acculturative stress refers to the stress that results from the process of adapting to a new culture. Berry (2006) coined the term “acculturation stress” to signify one's stress and one's reaction to the life events caused by acculturation. Studies have found that acculturation stress affects international students and has a positive relationship with mental disorders (Liu et al., 2022).

The literature focuses primarily on international students' distress as a response to the problems they encounter in the host country. It has been found that during the acculturation period, the most distressing adjustment problems for international students are homesickness, loneliness, loss of status or identity, difficulties in accessing support services, stereotyping, prejudice, racial discrimination, differences in social and cultural norms, financial problems, dietary restrictions, and language inadequacies (Wenhua and Zhe, 2013; Kristiana et al., 2022). Snoubar (2017) shows that international students are discriminated against by their peers mainly because of their language inadequacies, cultural differences, speaking in their native language, sexual orientation, political beliefs, skin color, religion, or race. A study conducted in Türkiye also points out the problems related to language inadequacy and accommodation problems, male–female relationships, and the differences in religious practices (Ilhan et al., 2012).

### 2.4. Coping strategies

Coping strategies refer to the various techniques individuals use to deal with stressors and challenging situations. Coping strategies are particularly relevant for international students who often face a variety of stressors, such as adjusting to new academic demands and dealing with social isolation. Recent literature has examined coping strategies and their association with various outcomes among international students. For example, one study found that international students who used emotion-focused coping strategies (such as seeking social support or using positive reframing) reported higher levels of psychological wellbeing and lower levels of stress (Chudzicka-Czupała et al., 2023). Another recent study examined mindfulness as a coping strategy for international students. Researchers found that higher levels of mindfulness were associated with lower levels of perceived stress and better academic performance (Poudel and Subedi, 2020). Another study examined the impact of coping strategies on academic adjustment among international students. Researchers found that problem-focused coping strategies, such as information seeking and planning, were positively associated with academic adjustment.

In contrast, avoidant coping strategies, such as denial and distraction, were negatively associated with academic adjustment (Rezaei et al., 2020). Individual differences may lead individuals to use different coping strategies to respond to a stressful situation. For example, a study conducted in Türkiye with African international students found that their main coping strategies were building networks with other Africans in the country, befriending Turkish people, learning Turkish, keeping in touch with their families, studying hard, being optimistic, and engaging in language practices (Musizvingoza, 2020). Although coping strategies help individuals cope with stressors, they can be adopted in adaptive or maladaptive ways. Adaptive coping strategies provide a relatively permanent solution to the problem without experiencing conflict or distress while maintaining wellbeing (Pearlin and Schooler, 1978). Maladaptive coping strategies, on the other hand, are helpful in the short term but have a negative long-term impact on the individuals' physical or psychological health and wellbeing (Stonham, 1996). The literature on the coping strategies of international students in Türkiye is rather limited. Therefore, the current study aimed to contribute to the literature by understanding the coping strategies of Arab female international university students when encountering stressors in Türkiye.

## 3. Methods

### 3.1. Procedure

This research consists of two parts. The first part aimed to discover the different adjustment domains of Arab female international university students. The second part aimed to explore their prearrival expectations, acculturation experiences related to adjustment issues, and coping strategies in response to the challenges they face in the domain where they meet the most challenges. The mixed methods research design was the most appropriate option for conducting this research. For the quantitative study, our null hypothesis is that the overall adjustment of Arab female international university students is equally influenced by each of the three adjustment domains. On the contrary, our alternative hypothesis is that overall adjustment is not equally influenced by the three adjustment domains, i.e., academic, sociocultural, and personal adjustment. Three different methods were used to analyze the quantitative study.

First, the correlation analysis between the three subdimensions (academic, sociocultural, and personal) and the overall adjustment was conducted to investigate the extent to which the four are correlated with each other. Second, a one-way ANOVA was used to test whether the mean adjustment of the three subdimensions is the same.

Third, the linear regression model was used to assess the impact of the three subdimensions on the overall adjustment. Linear regression also facilitated the investigation of the subdimension that would dominate the overall adjustment.

STATA was used for the quantitative analysis.

For the qualitative part of the research, interviews—consistent with the findings of the quantitative data—were conducted to have an in-depth understanding of the experiences of Arab female international university students in the areas that they found most challenging. The meaning-making process of qualitative data is mainly based on coding or categorizing the data, which involves three steps: reducing the significant volume of the raw data, identifying the critical patterns or themes of the data, and drawing conclusions (Wong, 2008). In the research analyses, the data turned to voice recordings in written form. Later, the open coding method was used to identify the themes. Open coding involves reading the raw data and dividing it into categories of information (Ozanne et al., 1992). The data were coded according to the common themes related to the three categories titled pre-arrival expectations, postarrival adjustment challenges, and coping strategies. Under each category, the common phrases were coded; for example, the theme of challenges due to cultural differences summarized several codes, including communication problems due to language issues, involvement problems due to sociocultural differences, and Arab–Turkish female cultural differences. The whole coding process was carried out using the qualitative data analysis software program MaxQDA10.

### 3.2. Participants

This research was conducted in two parts: quantitative and qualitative, and the samples were selected separately for both studies. For the quantitative part of the study, the researchers used a selective random sampling technique to adequately represent the Arab countries, and they randomly selected participants from each Arab country who were enrolled in bachelor's and master's programs at universities in the Republic of Türkiye. Participation in the research was voluntary, and the researchers approached the participants online or face-to-face at their respective campuses. Participants were selected from the six largest cities of Türkiye: Ankara, Antalya, Istanbul, Izmir, Konya, and Trabzon. These metropolitan cities include most of the universities in Türkiye and have the largest population of Arab female international students enrolled in their different universities. The average age of the bachelor's sample group of Arab female international university students was 20.5 years, and the average age of the master's sample group students was 26 years. The samples were drawn from both public and private universities. The sample size for quantitative data was 373, and the countries of the sample group are listed in Table 1.

**Table 1:** The sample size and the origin countries.

**Origin of country**	**Undergraduate**	**Graduate**	**No of students (*n* = 373)**
Algeria	10	5	15
Egypt	19	9	28
Iran	11	7	18
Iraq	12	6	18
Jordan	15	9	24
Lebanon	15	9	24
Libya	13	9	22
Morocco	13	9	22
Palestine	18	12	30
Saudi Arabia	18	10	28
Somalia	12	6	18
Sudan	11	5	16
Syria	20	11	31
Tunisia	16	9	25
United Arab Emirates	16	10	26
Yemen	16	12	28
Total			373

The study used one-on-one and group interviews to gain an in-depth understanding of the adjustment challenges. The researchers formulated open-ended questions after an extensive literature review, and the questions were prepared according to the needs of the research.

To have an equal representation of the sample from each Arab country in the qualitative part of the study, a selective random sampling method was used. To avoid any effect of the quantitative study, the participants of the qualitative study were 19 Arab female international university students enrolled in different universities in Türkiye who had not participated in the quantitative study. Their countries of origin are listed in Table 2. The age group of the study sample ranged from 18 to 30 years, and the average age was 20 and 23 years for the bachelor's and master's students, respectively.

**Table 2:** The sample size and their origin countries.

**Origin of country**	**Number of students (*n* = 19)**
Algeria	2
Egypt	2
Jordan	2
Lebanon	2
Palatine	2
Saudi Arabia	3
Somalia	2
Syria	2
Yemen	2
Total	19

### 3.3. Data collection

Quantitative data were collected using the International Students Adjustment Scale (Nazir and Öztürk, 2022). The scale can be administered to international university students at all levels, including bachelor's, master's, and doctoral students. It consists of 33 items ranging from “strongly agree” to “strongly disagree” and includes basic demographic information such as age, department, university, nationality, Turkish language proficiency, and the length of stay in Türkiye. On average, participants took 25 min to complete the scale. The reliability of the scale is 0.89, and the Kaiser–Meyer–Olkin (KMO) value is 0.80. It was developed in Türkiye and has been found to be highly reliable.

For the quantitative study, semistructured interviews with open-ended questions were used to collect data. The interviews were conducted one-on-one and in groups and were recorded. The researchers obtained written consent from the interviewee prior to recording, and they were assured of data protection. The interviews were conducted separately from the quantitative data collection process to avoid any influence on the qualitative findings.

## 4. Results and discussion

### 4.1. Quantitative data results

The pairwise correlations of the three subdimensions of adjustment and overall adjustment are presented in Table 3. Sociocultural and academic adjustment are correlated, as shown by the correlation coefficient of 0.40. Similarly, the sociocultural subdimension has the maximum correlation of 0.667 with overall adjustment. However, the other two subdimensions, academic and personal adjustment, are significantly correlated with overall adjustment with correlations of 0.642 and 0.552, respectively.

**Table 3:** Pairwise correlation.

**Variables**	**Academic adjustment**	**Sociocultural adjustment**	**Personal adjustment**	**Overall adjustment**
Academic adjustment	1.000			
Sociocultural adjustment	0.398^*^	1.000		
Personal adjustment	−0.144	0.005	1.000	
Overall adjustment	0.642^*^	0.667^*^	0.552^*^	1.000

^*^Significant when *p* < 0.05.

A one-way ANOVA was carried out to test whether the three means of the sub-dimensions of overall adjustment are the same against the alternative that at least two are different, and the results are presented in Table 4. The results show that the null hypothesis is rejected at a 1% significance level, as the *p*-value is approximately zero. This implies that the three means of the subdimensions of overall adjustment (academic, sociocultural, and personal) are different.

**Table 4:** One-way ANOVA.

**Source of variation**	**SS**	**df**	**MS**	**F**	***P*-value**	**F crit**
Between groups	4,600.427	2	2,300.213	91.749	0.0000	3.022362
Within groups	9,301.237	371	25.0707			
Total	13,901.66	373				

Multiple regression models with OLS estimates were used for further quantitative analysis. The results are presented in Table 5. Table 5 shows the influence of its various subdimensions, such as academic, sociocultural, and personal adjustment, on overall adjustment. After running the linear regression model and taking overall adjustment as the dependent variable, the academic, sociocultural, and personal adjustments were taken as independent variables. The result shows that all independent variables have a significant impact the dependent variable, as indicated by the high *R*^2^-value.

**Table 5:** The relationship between overall adjustments and three different subdimensions of adjustment.

**Overall adjustment**	**Coef**.	**St.Err**.	***t*-value**	***p*-value**	**[95% Conf**	**Interval]**	**Sig**
Academic adjustment	0.275	0.04	6.93	0.000	0.197	0.354	***
Sociocultural adjustment	0.347	0.046	7.57	0.000	0.256	0.438	***
Personal adjustment	0.253	0.06	3.84	0.000	0.201	0.363	***
*R* ^2^		0.612					
F-test		87.484		Prob > F		0.000	

The ^*^symbol indicates significance at *p* < 0.1. The ^**^symbol indicates significance at *p* < 0.05. The ^***^symbol indicates significance at *p* < 0.01.

Furthermore, social–cultural adjustment has a greater impact on overall adjustment than the other two dimensions. The highly significant value of the *F*-test indicates that the model is the best fit, and the three subdimensions are jointly significant determinants of overall adjustment. A unit change in academic adjustment will contribute 27.4% to the overall adjustment, and if there is a decrease in the academic adjustment, the overall adjustment will also decrease. Similarly, the personal adjustment would contribute 25.3% to the overall adjustment, and an increase in personal adjustment will also increase the overall adjustment. The sociocultural element is dominant in contributing positively to the overall adjustment, with a contribution of 34.7%.

### 4.2. Discussion of the quantitative study

To summarize quantitative analysis, the null hypothesis of equal means of the three sub-dimensions (academic, sociocultural, and personal) is rejected, which means that the contribution of each subdimension is not equal to the overall adjustment. To find out which subdimension contributes most to the overall adjustment among Arab female international university students, we conducted pairwise correlation and multiple regression analysis. Each analysis indicates that the sociocultural dimension contributes the most to the overall adjustment.

The results provide the answer to the research's first question, i.e., What is the most challenging adjustment domain for Arab female international university students in Türkiye?, and found that its sociocultural dimension is the most challenging domain for adjustment among Arab female international university students. The results also showed that the sociocultural dimension contribution is highest in the overall adjustment other than academic and personal dimensions.

The findings of the quantitative study are in line with the previous studies that the overall adjustment of international students is influenced by different domains (Zhang and Goodson, 2011; Lin et al., 2012; Wu et al., 2015; Alsahafi and Shin, 2019) and they can be broadly categorized into academic, sociocultural, and personal domains (Aderi et al., 2013). In addition, previous studies on Arab female international university students in the United States found that these female international students from the Arabian Gulf countries face more challenges in the sociocultural domain of adjustment (Shabeeb, 1996). The quantitative study results conclude that Arab female international university students face challenges in all three domains, i.e., academic, sociocultural, and personal domains. However, they face the most number of challenges in the sociocultural environment when it is compared to the other adjustment domains.

### 4.3. Qualitative data results

The qualitative data findings were examined in three categories according to the research questions.

#### 4.3.1. Prearrival expectancies

The participants expressed several prearrival expectations that primarily revolved around four key themes: quality of education, cultural similarity, political stability, and a substantial Muslim population. Every student interviewed stated that one of the main reasons for choosing Türkiye was their religious concerns. The students identified Türkiye as a unique location between the West and the East, which offered the advantage of receiving a quality education while ensuring safe religious practice. In addition to the common themes mentioned above, which acted as pull factors for choosing Türkiye for their education, students highlighted the push factors that strengthened their decision. For example, political instability in their home countries, deteriorating quality of education, limited educational opportunities, and the desire to practice religious freedom pushed them to seek educational opportunities in Türkiye. (Yemeni): I came to Türkiye with my family for education. We chose Türkiye because Turkish culture is like Yemen culture. Also, Muslims are safe in Türkiye. I could go to another Arab country (to practice my religion), but it is not so easy. Those countries are not politically safe.

(Algerian): My biggest motivation to study abroad was that the academia in my country is not that good, not that motivating to be there, to spend time there, to work on yourself, and to be an academic. Because it is a very low level, we do not have a good reputation in social sciences and humanities in my country (…). They do not really respect these sciences and the knowledge we could get is very poor, I can say (…). I had the option of the eastern and western sides of the world. I chose Türkiye because I wanted to travel at a very young age, I was 18, and Türkiye was safe for my religion and beliefs. An easier application and enrollment process was also an attractive option to study in Türkiye.
(Saudi Arabian): Saudi Arabia is a country where higher education is hard to get into. Especially in universities, you have to have at least a 95% GPA in high school. We have a Scholastic Aptitude Test (SAT) exam you have to get a very high score on it to get into universities. But in Türkiye, it was so much easier.

The contemporary portrayal of Turkish cinema and television series has captivated students, giving them the expectation of being able to practice their faith freely and giving them the feeling of being under the shadow of their religion, which is not the case in their home countries.

(Lebanese): I love Turkish dramas, and I used to watch them s[sic] lot before I came to Türkiye. In those shows, no one wears a hijab, no mosques are shown, and no one prays. Although I knew that the Muslim population in Türkiye was very high, I was afraid of being an outsider. But, when I arrived here, I saw all the beautiful mosques around me, heard the adhan (call to prayer) from everywhere, and felt comfortable.

#### 4.3.2. Postarrival adjustment issues

It is common for most of the students to face challenges in adjusting to life in Türkiye. In particular, Arab female international university students face common postarrival adjustment problems related to their cultural upbringing. Many of these students are not accustomed to gender-specific cultural differences and interactions with the opposite sex in their daily lives in their home countries. Their education in single-gender schools with limited contact with the opposite sex has made adjustment to the gender-mixed environment of Türkiye a source of anxiety and difficulty upon their arrival.

(Palestinian): I had never used public transportation before; it was a big change in my life. A man can tap you on the shoulder to tell you he wants to get off the bus. That is not something that can happen in my country.
(Yemeni): Buying groceries is a male task in our society, especially going to stores where men are the service providers. I had never done that before I came to Türkiye. In my first year, the day I had to buy medicine for my friend, I felt so scared, and cried. I avoided buying things as much as possible because I was not used to talking to the male pharmacist.

On the contrary, these students also faced a language barrier and discrimination from middle-aged and elderly Turks. Many were subjected to belittling and attempted deception and had to navigate the difficulties of being foreign women in Türkiye.

(Palestinian): In my first year, whenever I tried to say something and could not express myself, people would tell me “You have to learn the language if you are going to live in this country.” In addition, I completely refused to speak and learn this language (…). Because I had the belief that if I spoke in their language, they could benefit from me.
(Saudi Arabian): When taxi drivers understand that I am a foreigner, they double the price or take a longer route. When I tell them what they do, they get so angry. Then I feel so scared because I feel like they can do anything to me. So, I choose to be silent.
(Egyptian): I have no words for young Turkish men, they are very respectful and nice. But, old men can be very annoying. When they know that I am a foreigner, taxi drivers double the price. In addition, they have often asked for my number and even asked me to marry them. Even bus drivers do the same. When this happens, I pretend I don't understand and say “anlamadim” (I don't understand). They try to explain it repeatedly; I try to divert their attention because I am scared.
(Somali): Some men in Türkiye are so respectful to women, they help, but some of them make me feel so uncomfortable. While we are walking on the street, they are catcalling, especially in some places like *Aksaray* (an old tourist area in Istanbul). In addition, something happened once with [sic] taxi driver. He started asking questions like ‘Are you married?' It was none of his business, but I could not talk like that because I was afraid he might do something to me.

Several Syrian students noted that discrimination based on their country of origin is a common adjustment problem they face.

(Syrian): Once when I got a cab, I wanted to practice my Turkish and if you ask me, I was good. However, absolutely I have an accent and the driver asked me where I am from. When I said I am Syrian. He looked at me, with a shocked face and said “but you don't look dirty as Syrians do.
(Lebanese): When people hear my broken Turkish, because they think I am Syrian, they show negative facial expressions. But, when I tell people that I am Lebanese, they get so happy, and they start talking about how much they love our food.
(Saudi Arabian): Everyone has bad experiences with different cultures, and I have had many bad experiences with Turkish people. There is something traumatic that happened once. A Turkish woman accused my mother of hitting her car when she did not. So, she ended up physically hitting my mom and pulling the hijab off her head. Later she said that she thought that my mother was a Syrian.

#### 4.3.3. Coping strategies

Experiencing adjustment issues is a common occurrence among international students, and they often adopt different coping strategies. Similarly, Arab female international university students adopted common coping strategies to deal with distressing adjustment problems, such as making efforts to learn the Turkish language and avoiding areas where they faced discrimination. They preferred to live in areas with a significant Arab population, kept in touch with their families through phone calls, and maintained their religious practices through prayer.

(Saudi Arabian): I am also Syrian, on my mother's side. I can speak like a Syrian. I live in Başakşehir and we used to live in Fatih. When I speak with my Syrian accent, I feel at home in Türkiye.
(Yemeni): In Başakşehir, I do not feel the need to learn Turkish. Because I can use Arabic outside and I can speak English at the university. There is no place here where I need to use Turkish. When I go out of Başakşehir, I feel like I am leaving my home.
(Palestinian): I feel at home in Başakşehir. When I travel, I feel insecure and want to come back immediately.

As noted earlier, students use a variety of coping strategies to mitigate discrimination. For example, one student refuses to learn or speak Turkish because the locals judge her whenever she speaks because of her lack of proficiency in the language. In contrast, another student strives to become fluent in Turkish so that he/she may not be distinguished from the local Turks.

(Lebanese): We heard that Turkish people are so racist but, I realized that it is only for certain people. When people hear my broken Turkish because they think I am Syrian, their faces get an unpleasant mood. But when I tell people that I am Lebanese, they get so happy, start talking about how much they love our food. So, I will not speak at all until I learn Turkish very well because I do not want to be discriminated against.

Some students often use religious coping strategies to deal with negative experiences, finding solace in their beliefs and religious practices. In addition to the coping strategies mentioned above, they also reach out to friends and family back home to cope with the emotional distress caused by homesickness.

(Jordanian): The times when I struggle the most, I wake up in the middle of the night and pray; because I know that God is listening to me and that I am not alone here.
(Lebanese): In general, I do not feel homesick. I just miss my family. When I miss them, I call them and talk to them, but sometimes it is too late to call them. Also, when I feel sad, I cannot sleep. So, I listen to the Qur'an and pray. It gives me relief (…). Also, when I experience something bad, I counsel myself and say, “God has plans for you, trust His plan.”
(Yemeni): In Yemen, the first source is religion and science is not the absolute truth. Discovery in science is always presented in an integrated way with religion. In Türkiye, it is the opposite and I have found my religious explanations for science. I read more about religion and try [sic] to integrate my studies with religion.

### 4.4. Discussion of the qualitative study

#### 4.4.1. Prearrival expectancies

Prearrival is the period prior to travel to the host country. It has been identified as the predeparture period and the stage for “entry points” of study abroad (Durkin, 2008). This phase of the international student's journey has not been the focus of the studies earlier. However, it is one of the crucial stages that needs to be understood and plays an essential role in the overall adjustment.

The study used qualitative methods and found that several factors influenced the Arab female international university student's prearrival expectations in choosing Türkiye for their education. These factors included the quality of education, cultural similarity, political stability, and the large Muslim population. The students perceived Türkiye as a unique location between the West and the East that would allow them to receive a quality education while also practicing their religion safely.

Studies have suggested that Muslim students tend to choose a Muslim host country for their education (Çetin et al., 2017). Our research findings support this claim, as every student interviewed stated that one of the main reasons for choosing Türkiye was their religious concerns. Although some students were hesitant about the secularity of Türkiye, these concerns disappeared upon their arrival.

Many scholars (Ilhan et al., 2012; Özoglu et al., 2015; Snoubar, 2017; Aydin, 2021b) have already demonstrated the importance of quality education, political stability, affordability of living, recommendations from others, geographical proximity, cultural similarity, and scholarship opportunities as pull factors for international students to choose Türkiye as a destination country. Our findings are consistent with earlier studies on this point. In addition, our participants also mentioned the country's natural scenery, similar climate, safer environment, easy access to clean water and electricity, and better economic conditions through dormitories and scholarship opportunities as pull factors during the interviews.

#### 4.4.2. Postarrival adjustment issues

The postarrival adjustment challenges examined the problems faced by Arab female international university students in adjusting to their new environment upon arrival. The results showed that, although the students initially believed that Turkish culture was similar to theirs, they discovered differences as they experienced the culture and daily life in Türkiye. For example, students from Palestine, Jordan, Saudi Arabia, and Yemen had never used public transportation before and found it to be an incredible experience. It is important to clarify that the students had not used public transportation before because they were not as wealthy in their countries. However, culturally, it is not appropriate for women to use public transportation alone without being accompanied by a man, which is related to religious concerns. In addition, not only public transportation but also grocery shopping or going to the pharmacy was a new experience for some students.

As explained earlier, when they had to shop for their needs, they faced difficulties for two reasons: first, it is considered a man's work in their country, and second, they were not much exposed to an environment with strangers of the opposite sex.

Another typical problem that students face is discrimination. They reported that they did not face discrimination from the younger generation but from middle-aged and older Turkish people. The most common reason for discrimination was their lack of language skills; they were belittled and exploited. In addition, they had to deal with the difficulties of being a foreign woman in Türkiye.

In this case, a student admitted that she was too tired of being underestimated or belittled because of her language skills. The forceful statements concerning the need to learn the language may have caused her to have negative feelings and thoughts related to Turkish. If she learned the language, the people who forced her would be able to communicate with her comfortably, but she would be the one struggling with the challenges of learning the language. Her words can be interpreted in many ways, and she may be trying to cover up her belief that she is “incompetent in language learning” by saying she refuses to learn the language.

Parallel to the literature, Syrian students talked about their experiences of discrimination. In contrast, students from other countries stated that they were discriminated because Turkish people assumed that they were Syrian. Discrimination is not general but mainly against Syrians. Although our research did not aim to collect data on discrimination rates, the interviews illustrate that Turkish people can be verbally, and sometimes physically, harsh toward foreigners. The increasing number of refugees and the debates surrounding them have created a negative perception of Syrians in Türkiye (Arslan et al., 2017). Kaya and Kiraç (2016) revealed many experiences of Syrian refugees related to hostility, bullying, and discrimination in Turkish society.

The students mentioned other experiences that they found shocking or stressful. For example, three students admitted that it was surprising for them to see a hijab-wearing woman smoking. Open alcohol consumption in a country with a large Muslim population was also new to some students. Students from Yemen, Palestine, Jordan, Lebanon, and Saudi Arabia admitted that seeing men and women in intimate ways in public places, especially women with hijabs, was something they were not used to in their home country.

The research results on the challenges of adjustment faced by Arab female international university students after their arrival in Türkiye revealed significant cultural differences despite sharing the same religion and gender equity, especially in everyday tasks. Students also have limited exposure to the opposite sex outside of their homes and face discrimination from specific segments of the population, as well as due to their lack of proficiency in the Turkish language and because of their religious practices or attire. Although there is limited literature focusing on the adjustment challenges faced by Arab female international university students, studies have found similar results. For example, a study conducted in the United Kingdom focused on Omani students and examined the challenges faced by Arab female international university students when studying abroad. The study highlighted significant cultural differences, limited exposure to the opposite gender outside their homes, and discriminatory attitudes and behaviors that these students face in the United Kingdom (Al Weshahi, 2022). Similarly, a study of Saudi Arabian female students in the United States found that these students face significant cultural and gender role differences, particularly in the workplace. The study also found that they have limited exposure to the opposite gender outside of their home countries, which makes the sociocultural environment of the host country challenging, and that they face discrimination based on their religious attire (Arafeh, 2020). Studies on Syrian students have concluded that refugees face discriminatory attitudes in Turkish higher education institutions (Özenç-Ira et al., 2023). Thus, it can be concluded that Arab female international university students face numerous adjustment challenges in the sociocultural domain upon arrival, which affect their overall adjustment, in line with the available literature.

#### 4.4.3. Coping strategies

As students go through the process of acculturation, they tend to develop coping strategies that are either adaptive or maladaptive. These strategies are used to deal with various negative experiences, such as discrimination, cultural differences, conflicting preferences, unexpected behaviors or attitudes of local people, and differences in religious practices. For example, most of the students interviewed chose to visit only the neighborhoods with a large Arab population, such as Başakşehir (the most Arab-populated area in Istanbul) and Fatih (the tourist center of Istanbul). Previous research has shown that avoidance is a commonly used coping strategy among students, which supports the findings of this study (Wei et al., 2007; Amponsah, 2010). Furthermore, it has been observed that students use different coping mechanisms to avoid experiencing discrimination. For example, as mentioned earlier, one student chose not to learn or speak Turkish to avoid being judged by locals whenever she made a mistake. Meanwhile, another student aims to become proficient in Turkish to fit in and not be distinguished from the local Turks. The use of different coping strategies by these students highlights the importance of individual differences. Even when faced with similar situations, the two students responded differently because of their unique intentions. In addition, Arab female international university students are often observed to rely on religious coping strategies to deal with negative experiences. Their religious beliefs and practices serve as a source of support during difficult times. This finding is supported by previous research that indicates that, during times of stress, female students tend to use coping strategies that include focusing on and expressing their emotions, seeking emotional and social support, and relying on religious coping techniques (Sapranaviciute et al., 2011).

Apart from conventional coping strategies, individuals also resort to unique coping methods that vary according to personal differences. For example, some may change their environment to reduce negative energy, seek relaxation by going to the beach, engage in activities such as walking and dancing, or try to understand the underlying dynamics of the problem rather than avoid it. While employing task-oriented strategies to cope with stressors may be considered a more effective overall approach, engaging in purposeful distracting activities may provide brief respite and momentary relaxation, as they may temporarily relieve highly stressful emotions, thereby enhancing psychological wellbeing (Hahn, 2010). In addition, specific maladaptive emotion-focused coping strategies were commonly observed among students, such as distracting attention from the problem, avoiding going out, avoiding social interactions, oversleeping, and crying. These findings are consistent with previous studies showing that students commonly use coping strategies such as avoidance behavior, oversleeping, and emotional responses like crying (Amponsah, 2010; McDermott et al., 2021).

It was observed that most students were often reluctant to seek help or discuss their problems with others. Despite the availability of free counseling services provided by the university, the majority of students did not use them. Of the 19 students interviewed, only four mentioned seeking help or talking to someone concerning their problems. As a result, most of the students' coping strategies were solitary activities. While recognizing and acknowledging emotional distress may be beneficial for some individuals in managing negative feelings (Endler and Parker, 2008), it may not be helpful for some international students due to their cultural tendency toward emotional reserve and reluctance to express emotions (Kim et al., 1999). Emotional self-control is often considered an essential cultural value and a symbol of maturity in some cultures (Kim et al., 2005).

The research showed that Arab female international university students used different coping strategies based on their individual and cultural differences.

## 5. Conclusion

The current research focused on the adjustment experiences of Arab female international university students in Türkiye. Both quantitative and qualitative methods were used in the study. The quantitative study aimed to reveal which adjustment domain is the most challenging for Arab female international university students in Türkiye. The results showed three challenging environments: personal, academic, and sociocultural; among them, students experience more challenging problems in the sociocultural domain of adjustment. Based on the quantitative results, the researchers attempted to explore students' experiences in the sociocultural environment more deeply by using semistructured qualitative interviews. During the interviews, the researchers aimed to discover the students' prearrival expectations, postarrival adjustment challenges, and the coping strategies they used to deal with the challenging stressors.

The prearrival expectations include the push and pull factors, such as students' motivations for choosing Türkiye as a destination country for their studies. The results of the qualitative study were in line with the existing literature. Moreover, quality of education, recommendations of friends, religious concerns, geographical proximity, cultural similarities, mild weather conditions, political stability, easy access to resources, better economic conditions, scholarship and housing opportunities, political instability in their home countries, deteriorating quality of education, limited educational opportunities, and the desire to practice religious freedom were the push and pull factors for students to decide to study in Türkiye.

In addition to understanding their prearrival motivations, the research aimed to discover their postarrival challenges. It was found that, although most of the students said that they mainly found what they expected, some even admit that their hesitations disappeared after coming to Türkiye. They also had negative experiences that caused stress during acculturation and adjustment. Using public transportation, interacting more often with male colleagues, dealing with work that is considered male work in their country, discrimination, inappropriate suggestions, Turkish people's curiosity about their private lives, and daily practices of Turkish people such as smoking, drinking, intimate relations in public, differences in religious practices, and the taste of the food were the things they mentioned as the most difficult aspects to adapt to.

Finally, the study aimed to investigate students' coping strategies to deal with stressful events and negative experiences during their adjustment process. It is found that there are personal differences in coping strategies to deal with the same situation. For example, to avoid discrimination, one student prefers not to speak Turkish and he/she avoids learning it; conversely, another student aims to learn Turkish so well that no one can understand that she is a foreigner and discriminate against her. Engaging more in religious practices, calling friends and family members back home, wearing traditional clothes, and cooking traditional food were common ways of coping with stress. In addition, some students prefer to think about the problem to understand its dynamics, go for a walk, or dance to feel better and relax. The enlightening part of the study is that they found out that the coping strategies were generally things that the students could do themselves. It is noted that asking others for help does not seem to be a common way for these students to deal with the stressors. It is concluded that students deal with stress by avoiding it or facing it, but almost always alone. However, seeking professional help can be crucial in the adjustment process (Chudzicka-Czupała et al., 2023). If counseling services were promoted and shown to be effective, more students might consider benefiting from them instead of relying on maladaptive coping strategies that can lead to experiencing depression and anxiety.

This study significantly contributes to the literature by examining a minority group from a gender perspective. The findings highlight the importance of raising awareness among students about studying in a different country and culture. More importantly, the study shows that the population is reluctant to seek help dealing with stressors. Therefore, mental health professionals can play an important role in encouraging international students to seek help and guiding them in finding adaptive ways to cope with stressors. There is a need for orientation programs and student support systems that students can trust and share their experiences and problems with. Students should be prepared for cultural differences before they arrive so that they are satisfied with their higher expectations. Psychoeducation can help students understand and accept differences and protect their mental health when facing difficult situations (Elemo and Türküm, 2019). Türkiye has made great strides in becoming a destination country for education. However, there are still necessary steps to be taken. Universities should improve the services of their international offices and provide psychoeducation to their international students with the help of counselors and psychologists. Mental health professionals should be aware of cultural differences and improve their skills in fostering multicultural environments. Researchers can work to implement effective orientation programs that inform and encourage the use of psychological support systems.

The present research has limitations, including a small sample size in its qualitative study and limited coverage of major Turkish cities. Further studies can have a large sample group and include individuals from all regions of Türkiye.

## Data availability statement

The raw data supporting the conclusions of this article will be made available by the authors, without undue reservation.

## Ethics statement

The studies involving human participants were reviewed and approved by Ibn Haldun University Ethical Committee board. The patients/participants provided their written informed consent to participate in this study.

## Author contributions

TN and AÖ contributed to the conception and design of the study and wrote sections of the manuscript. TN organized the database and performed the statistical analysis. AÖ wrote the first draft of the manuscript. All authors contributed to the manuscript revision, read, and approved the submitted version.
